# Carbon Adsorbents from Spent Coffee for Removal of Methylene Blue and Methyl Orange from Water

**DOI:** 10.3390/ma14143996

**Published:** 2021-07-16

**Authors:** Inga Block, Christina Günter, Alysson Duarte Rodrigues, Silvia Paasch, Peter Hesemann, Andreas Taubert

**Affiliations:** 1 Institut für Chemie, Universität Potsdam, Karl-Liebknecht-Straße 24-25, D-14476 Potsdam, Germany; iblock@uni-potsdam.de; 2 Institut für Geowissenschaften, Universität Potsdam, Karl-Liebknecht-Straße 24-25, D-14476 Potsdam, Germany; guenter@geo.uni-potsdam.de; 3 ICGM, Univ Montpellier-CNRS-ENSCM, 34090 Montpellier, France; alysson.duarte-rodrigues@umontpellier.fr (A.D.R.); peter.hesemann@umontpellier.fr (P.H.); 4 Professur für Bioanalytische Chemie, TU Dresden, Bergstraße 66, D-01062 Dresden, Germany; silvia.paasch@tu-dresden.de

**Keywords:** water, spent coffee, dye adsorption, methylene blue, methyl orange, calcium carbonate, activated carbon, water treatment, dye removal

## Abstract

Activated carbons (ACs) were prepared from dried spent coffee (SCD), a biological waste product, to produce adsorbents for methylene blue (MB) and methyl orange (MO) from aqueous solution. Pre-pyrolysis activation of SCD was achieved via treatment of the SCD with aqueous sodium hydroxide solutions at 90 °C. Pyrolysis of the pretreated SCD at 500 °C for 1 h produced powders with typical characteristics of AC suitable and effective for dye adsorption. As an alternative to the rather harsh base treatment, calcium carbonate powder, a very common and abundant resource, was also studied as an activator. Mixtures of SCD and CaCO_3_ (1:1 *w*/*w*) yielded effective ACs for MO and MB removal upon pyrolysis needing only small amounts of AC to clear the solutions. A selectivity of the adsorption process toward anionic (MO) or cationic (MB) dyes was not observed.

## 1. Introduction

Water is among the most valuable resources worldwide. As a result, reliable methods for water treatment and remediation are among the most pressing issues worldwide. In addition to natural organic matter, heavy metals, biological contaminants, and disinfection byproducts are among the key pollutants in surface and drinking water. Synthetic organic substances such as dyes, oils, or pharmaceuticals and their metabolites or degradation products also play a major role in water pollution [1].

Many of these substances can be removed from the aqueous phase by activated carbon (AC) adsorbents. AC can be produced on an industrial scale from sources such as coal, petroleum, or coconut husk [2,3]. As a general requirement, the starting material needs to have a high carbon content, and many renewable raw materials are therefore candidates for AC production. Typical ACs have large surface areas between 500 and 1500 m^2^/g and pore volumes of up to 1.8 cm^3^/g [1]. Therefore, many groups have studied a diverse pool of natural (waste) products such as coconut shells, fruit seeds (grapes, pomegranate, date palm), olive and peach stones, wood wastes, pine cones, and more for the fabrication of ACs [4,5,6,7,8,9,10,11,12,13,14,15,16,17,18,19,20]. Other studies include composites of clay and carbonaceous materials [6,21,22,23]. For example, Okman and Karagöz et al. [10] chemically activated grape seeds with potassium carbonate and potassium hydroxide before carbonization between 600 and 800 °C, resulting in materials with BET surface areas of up to 1238 m^2^/g (K_2_CO_3_) and 1222 m^2^/g (KOH).

Dried spent coffee (SCD) is yet another interesting starting material for AC production. In Germany alone, every coffee drinker consumes about 3.6 cups every day [24]. Accordingly, large amounts of SCD are produced but are typically discarded. This is clearly a waste of raw material. Indeed, because very large amounts of SCD are available worldwide, SCD-based adsorbents have become a subject of intense research and development. SCD has been studied both in its parent, untreated form [3,25,26,27,28,29,30] and in the form of ACs made from SCD [31,32,33,34,35]. Typical procedures involve the impregnation of SCD with ZnCl_2_ and H_3_PO_4_ or treatments with NaOH before carbonization. The resulting ACs exhibit high porosities and large surface areas, along with functional groups on the AC surface. These factors are beneficial to the overall performance of the AC adsorbents.

For example, Alves et al. [35] described the synthesis of AC via the impregnation of SCD with ZnCl_2_ for 7 h at 85 °C before pyrolyzing this mixture for 2 h at 500 °C under nitrogen. Afterward, the remaining ZnCl_2_ was removed with 0.1 M HCl. The resulting AC had a BET surface area of 1039 m^2^/g and a total pore volume of 0.481 cm^3^/g. This is close to the total surface areas in commercial ACs. The carbon material had a maximum adsorption capacity of 123.22 mg/g for bisphenol A (BPA). In a related approach, Lamine et al. [34] activated SCD using a phosphoric acid treatment before pyrolysis at 600 to 700 °C for 1 h. The materials had surface areas up to 186 m^2^/g and phenol adsorption capacities up to 56 mg/g.

The current study describes the activation of SCD with sodium hydroxide. Moreover, the effect of calcium carbonate powder added to the raw SCD is also presented. Depending on the pretreatment, ACs with different properties (surface areas, surface functional groups, adsorption behavior) were obtained. Both activation processes led to materials that almost completely remove methylene blue (MB) and methyl orange (MO) from aqueous solution. These experiments include a first study on the kinetics, as well as a first study on the adsorbent dose dependence of the dye removal process. With increasing pyrolysis temperature, the effectiveness of the ACs increased. The approach of SCD activation with CaCO_3_ is a new and very low-cost, yet highly promising approach towards cheap adsorbents based on food waste (SCD) and a mineral easily found in nature (CaCO_3_).

## 2. Materials and Methods

### 2.1. Materials

Spent coffee powder was obtained from DEK Deutsche Extrakt Kaffee GmbH, Berlin, Germany. Ethanol (>96%, Carl Roth GmbH + Co. Kg, Karlsruhe, Germany), sulfuric acid (95–98%, Acros, Fairlawn, NJ, USA), hydrochloric acid (37%, Fisher Scientific GmbH, Wohlen, Switzerland), sodium hydroxide (>99.9998% Merck KGaA, Darmstadt, Germany), methyl orange (100%, Merck KGaA, Darmstadt, Germany), methylene blue (100%, AppliChem GmbH, Darmstadt, Germany), and calcium carbonate powder (≥95%, VWR, Darmstadt, Germany) were used as received.

### 2.2. Methods

*CHNS analysis* was carried out on an Elementar Vario EL III (Langenselbold, Germany) in duplicate.

*Thermogravimetric analysis* was done on a Linseis TGA/DTA L81 (Selb, Germany) from 25 to 1000 °C under nitrogen with a heating rate of 10 °C.

*FT-Infrared spectroscopy* was done on pulverized samples on a Nicolet iS5 (Thermo Scientific, Waltham, MA, USA, iD7 ATR unit with a diamond crystal, resolution of 4 cm^−1^, 32 scans, from 400 to 4000 cm^−1^).

*Scanning electron microscopy* (SEM) was done on a JEOL JSM-6510 SEM (Freising, Germany) with a tungsten cathode operated at 15 kV. Prior to imaging, all samples were sputter-coated with Au/Pd for 75 s and 18 mA using a SC7620 mini sputter-coater (Quorum Technologies, Lewes, UK).

*X-ray powder diffraction* data were collected on a PANalytical (Malvern, UK) Empyrean powder X-ray diffractometer in a Bragg–Brentano geometry equipped with a PIXcel1D detector using Cu Kα radiation (λ = 1.5419 Å) operating at 40 kV and 40 mA; θ/θ scans were run from 4–70° 2θ with a step size of 0.0131° and a sample rotation time of 1 s. The diffractometer was configured with a programmable divergence and anti-scatter slit and a large Ni-beta filter. The detector was set to continuous mode with an active length of 3.0061°.

*The specific surface areas* and pore characteristics were determined via nitrogen sorption measurements on a Micromeritics Tristar unit (Norcross, GA, USA) at 77 K. Prior to the experiment, each sample was degassed to about 2 Pa at 353 K for 10 h. The specific surface area (SSA) was calculated using the Brunauer–Emmett–Teller method (BET). The average pore sizes were estimated from the adsorption branch of the isotherm using the Barrett–Joyner–Halenda method (BJH). The pore volume was determined at P/P_0_ > 0.99.

*UV/Vis spectroscopy* was done on a Shimadzu UV-1900 (Kyoto, Japan) between 250 and 1000 nm with a sampling interval of 1.0 nm at medium speed in single-scan mode. Solutions were placed in PMMA cuvettes with a path length of 10 mm using water as a reference. All data were evaluated using Origin 2020b Pro.

*Solid state NMR spectroscopy*. ^13^C CP solid-state NMR spectra were recorded on a Bruker Avance 300 MHz spectrometer (Billerica, MA, USA) using a commercial 2.5 mm MAS NMR probe and operating at a resonance frequency of 75.48 MHz. The MAS frequency was 15 kHz. Chemical shifts are referenced to TMS using adamantane. Ramped ^1^H–^13^C cross-polarization (CP, contact time: 4 ms) and SPINAL ^1^H-decoupling were applied during the signal acquisition. The recycle delay was 3 s, and 24,000 scans (SCD) or 32,000 scans (PSC50) were accumulated.

### 2.3. Sample Preparation

**Figure A1:**
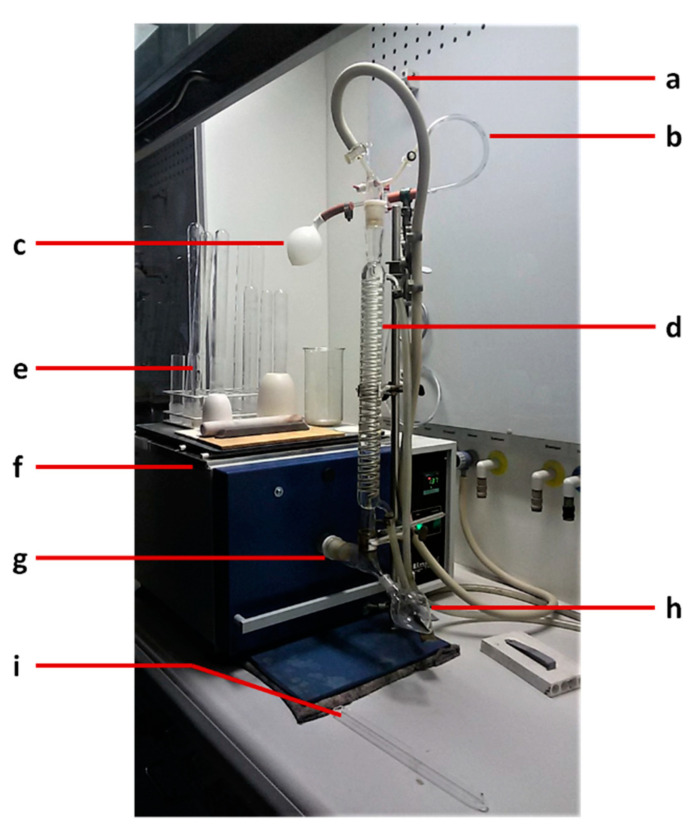
Customized pyrolysis oven system: (**a**) vacuum connection, (**b**) N_2_-line, (**c**) balloon as pressure relief valve, (**d**) spiral cooler, (**e**) fused silica glass tubes, (**f**) oven, (**g**) opening for fused silica glass tube insertion, (**h**) flask for condensation products, and (**i**) sample holder (fused silica glass).

Table 1 summarizes all materials studied. Firstly, as a control material, dried spent coffee (SCD) was pyrolyzed at 500 °C (PSC50). Secondly, the carbon precursor material Na90 was produced by pretreating SCD with NaOH for 24 h at 90 °C. Afterward, these solids were dried without filtration (these materials could not be filtered due to severe clogging of the filters). After drying, the powder was inserted into the pyrolysis oven (a custom modified oven system with an opening to insert and remove the fused silica glass reaction tubing; see Appendix A, Figure A1), and then the oven was heated to 500 °C at 5 °C/min. After 1 h at 500 °C the oven was switched off and allowed to cool to room temperature. After removal from the oven, the carbonaceous material was washed with 2 M HCl to remove residual NaOH from the powder to yield the pure carbonaceous material PNa90 and then washed to neutrality with d.i. water.

A second type of ACs was made via mixing SC with CaCO_3_ powder (weight ratio 1:1) and subsequent pyrolysis of the mixture between 500 and 850 °C for 1 h, again using a heating rate of 5 °C/min. Afterward, the resulting black solids were washed with 2 M HCl to remove the CaCO_3_ until the solution was acidic, followed by washing with d.i. water until neutral and drying. Lastly, all materials were hand-ground in a mortar to reduce particle sizes. Table 1 summarizes all powders studied in this work. These materials are designated as SCC## (SC + CaCO_3_) with the index ## indicating the pyrolysis temperature (Table 1). For example, SCC85 was made from a 1:1 mixture of SCD and CaCO_3_ pyrolyzed at 850 °C for 1 h.

### 2.4. Adsorption Experiments

In a first study, 100 mg of each material (Table 1) was dispersed in 10 mL of dye solution (23 mg/L of either MO or MB to accommodate the detection limit of the UV/Vis spectrometer) and shaken on an IKA KS 260 basic shaker at a 300 rpm shaking speed for 3 h at room temperature. Dye uptake of the solutions after filtering the solutions through PTFE syringe filters (VWR, 0.45 µm) was quantified with UV/Vis spectroscopy between 250 and 1000 nm (MO @ 461 nm, MB @ 664 nm).

All materials showing a measurable dye uptake were then studied for their adsorption kinetics at room temperature. To that end, 100 mg of adsorbent were dispersed in 10 mL of dye solution (23 mg/L) and shaken at 300 rpm for 1, 5, 30, and 60 min, and 3, 24, and 48 h, counting from the point of dye addition to the adsorbents. Additionally, MO uptake by PNa90 was studied for up to 2 weeks to examine if an equilibrium stage is reached.

A further series of experiments was used to evaluate the dose dependency of SCC85 on dye adsorption for MB and MO solutions, as well as on a MO/MB mixture. The mixture was made by mixing equal volumes of the MO and the MB solutions. Here, amounts between 1 mg and 80 mg of material were exposed to 10 mL of dye solution and shaken for 3 h at a 300 rpm shaking speed at room temperature. Again, the solutions were filtered through PTFE syringe filters afterward and then quantified using UV/Vis spectroscopy.

*Note*: A dye concentration of 23 mg/L was used in this study. The reason for selecting an MO and MB concentration of 23 mg/L is that this concentration provides good UV/Vis absorption data, while it also enables the measurement of very low concentrations. Higher concentrations produce too strong absorption signals in the spectra of MO, while lower concentrations produce absorption signals that are too weak to analyze in the MB spectra. Further work is aimed at investigating different dyes, different concentrations, and different conditions such as pH, T, and salt effects on the performance of these materials.

## 3. Results

In a series of preliminary experiments using all materials listed in Table 1, only two groups of materials remove the organic dyes methyl orange (MO) or methylene blue (MB) from aqueous solution. Pyrolysis of (1) pure SC (PSC50) and (2) SC with calcium carbonate at 500 °C (SCC50) leads to powders with low dye adsorption. These two products were therefore not considered for extended adsorption experiments.

All other materials were further investigated for their dye removal capacities. Table 2 shows the elemental analysis data of these materials along with the data obtained for the pure (non-pyrolyzed) SCD raw material. After pyrolysis, all powders show a high carbon content and rather small fractions of other elements. This confirms a successful carbonization process in all cases.

**Figure A2:**
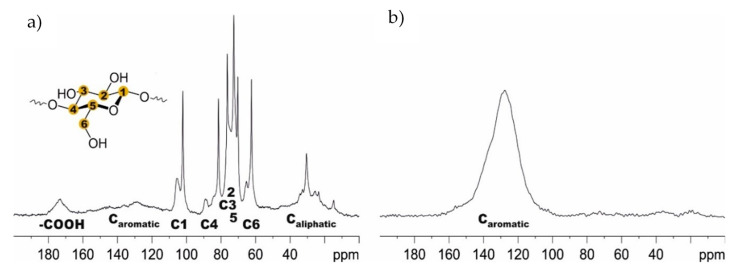
^13^C CP NMR spectra of (**a**) spent coffee SC and (**b**) PSC50.

Solid-state NMR (ssNMR; see Figure A2, Appendix A) investigations of SCD and PSC50 reveal the drastic change in material and structural properties upon pyrolysis. The comparison of ^13^C CP NMR spectra of the same material before (SCD, Figure A2a) and after pyrolysis (PSC50, Figure A2b) shows the influence of the temperature treatment. In the spectrum of raw SCD, well-resolved signals corresponding to all organic matter present in SCD such as cellulose, hemicellulose, and lignin, as well as proteins and lipids, can be observed [36,37,38,39,40]. Carbons of different polysaccharides such as cellulose and hemicellulose cause the intense and narrow signals between 60 and 110 ppm (Figure A2a). In the aliphatic region between 20 and 40 ppm, signals of lipids and protein side-chains appear. The broad signals between 120 and 160 ppm are probably due to lignin aromatic carbons, and the signal at 173 ppm can be assigned to carbonyl groups of lignin, hemicellulose, and proteins.

In contrast, after pyrolysis of this material, a broad signal at 128 ppm dominates the NMR spectrum. This signal can be assigned to highly condensed aromatic carbon atoms from carbon black. Furthermore, a shoulder at ca. 150 ppm is caused by phenolic carbons [41]. Very weak signals in the aliphatic region (20–40 ppm) are due to residual side-chains (Figure A2b). Solid-state NMR spectroscopy, therefore, clearly shows that the vast majority of organic building blocks in the raw SCD are broken down during pyrolysis and form condensed aromatic carbons afterward.

### 3.1. Activated Carbons from Chemically Pretreated Spent Coffee

**Figure 1:**
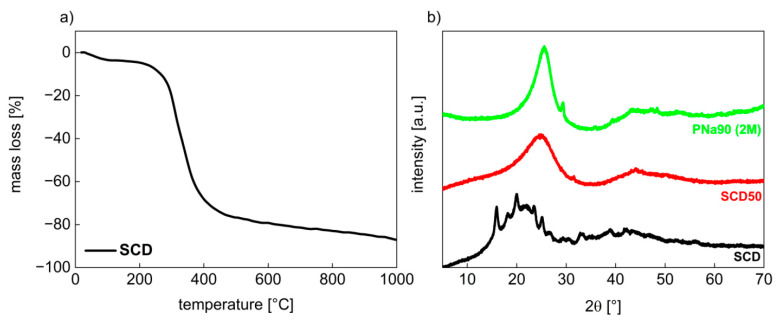
(**a**) Thermogravimetric analysis data of spent coffee (SCD). (**b**) X-ray diffractograms of 500 °C (PSC50) and pyrolyzed SCD pretreated with 2 M NaOH at 90 °C and washed neutral after pyrolysis (PNa90 (2 M)).

Figure 1a shows a representative thermogravimetric analysis (TGA) curve of SCD. A small step (5.7% weight loss) is visible at around 100 °C. This step is likely due to evaporation of residual water. A second and rather broad step (70.9% weight loss) is observed between 200 and 450 °C. This step can be assigned to the slow decomposition of hemicellulose, cellulose, and lignin (in that order), the main components of spent coffee [35,40,42,43,44]. Subsequently, the TGA data indicate a continuous weight loss until 1000 °C showing the decomposition of the sample [40].

Figure 1b shows representative X-ray diffraction (XRD) data of selected powders. XRD patterns of dried spent coffee (SCD) show two broad halos along with a series of reflections sitting on these halos (Figure 1b). Such a pattern is typical for cellulosic materials [44]. After pyrolysis, the sharp diffraction rays are not observed anymore and only two broad halos remain. These reflections are typical for graphitic carbon-type materials and can be assigned to the carbon (002) and (100) planes [35,45]. Occasionally, the XRD patterns show a few remains of the sharp reflections, but this is not always the case.

**Figure 2:**
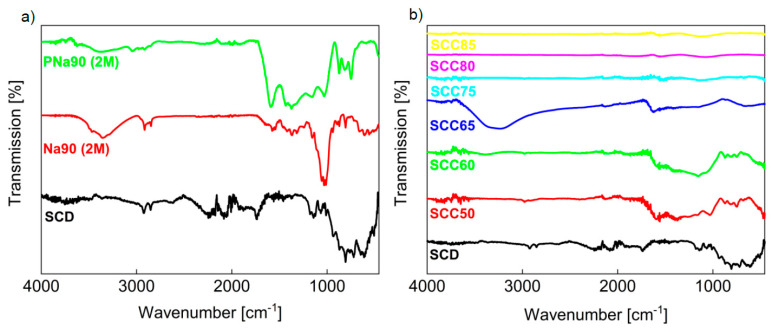
FTIR spectra of (**a**) SCD treated with NaOH before (Na90 (2 M)) and after (PNa90 (2 M)) pyrolysis, and (**b**) SC + CaCO_3_ pyrolyzed at different temperatures, all in direct comparison to dried SC (SCD). All bands can be assigned to the cellulose and lignin present in the materials [35,40,44,46,47,48,49] (Table 3). Bands in the area of 2000 cm^−1^ represent the natural resonance of the diamond crystal used for measuring.

Figure 2 shows the infrared (IR) spectra of SCD and of selected treated materials. The IR spectrum of the starting material SCD shows strong bands between 1000 and 500 cm^−1^ and a series of weaker bands at around 1700 and 2900 cm^−1^. After the pretreatment with NaOH and before pyrolysis, the bands in the fingerprint area are weaker than before. The bands at around 2900 cm^−1^ are still visible, and additional new bands between 1700 and 1000 cm^−1^ and at ca. 3400 cm^−1^ appear (Figure 2a). All bands can be assigned to the cellulose and lignin present in the materials [35,40,44,46,47,48,49] (Table 3).

After pyrolysis (Figure 2b) the bands between 1500 and 1000 cm^−1^ are broader and show a significantly increased intensity, while bands between 1000 and 500 cm^−1^ lose some intensity. No further changes can be observed in the IR data. While the ssNMR spectra of pure pyrolyzed SC (Figure A2) shows that the organic material was reduced to highly condensed aromatic carbon atoms, a treatment with NaOH beforehand seems to leave some functional groups on the material surface, which can be confirmed via ATR-IR.

**Figure 3:**
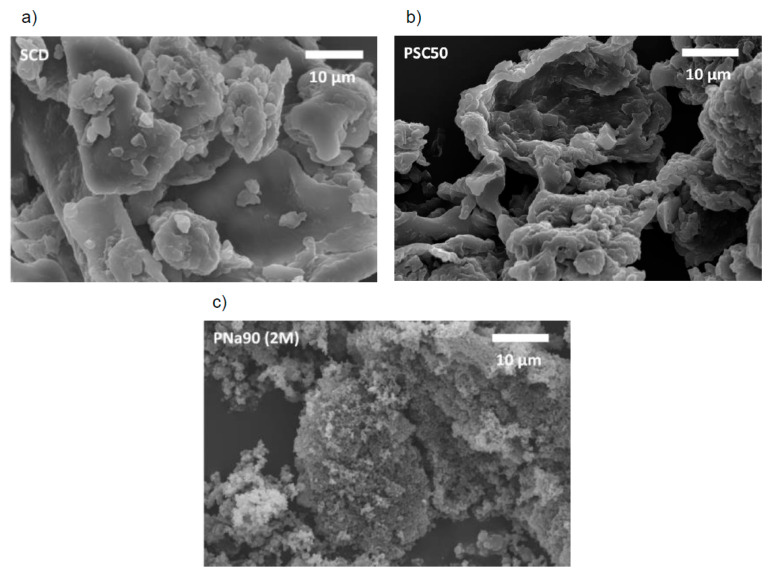
SEM images of (**a**) SC dried (SCD), (**b**) SC pyrolized at 500 °C and (**c**) SC treated with 2 M NaOH and py-rolized at 500 °C.

Figure 3 shows scanning electron microscopy (SEM) images of SCD after different treatments. The SCD material is rather heterogeneous in terms of both the particle size and the particle shape. There are elongated and rather thin particles with lengths reaching up to 100 µm, along with rounded particles, and numerous aggregated particles of various sizes and shapes. The surfaces of these particles are smooth and without any holes (Figure 3a). Pyrolysis of pure SCD at 500 °C has no obvious effect on the surface of the resulting carbons (Figure 3b).

**Figure A3:**
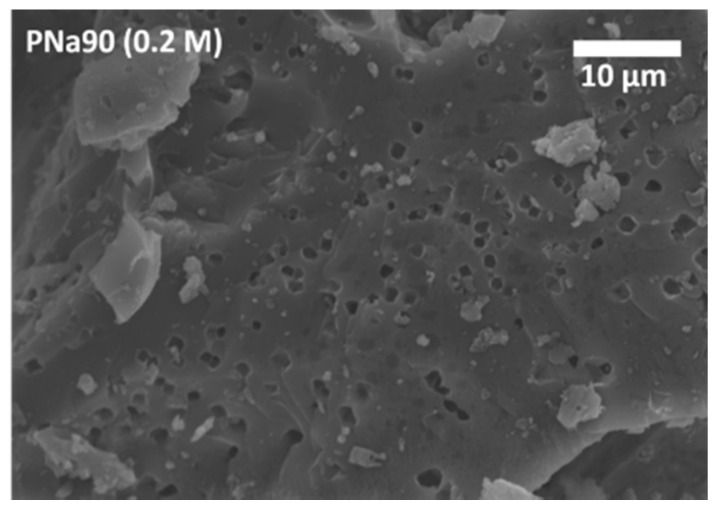
SEM image of SC pretreated with 0.2 M NaOH at 90 °C after pyrolysis at 500 °C.

Pretreatment with 2 M aqueous NaOH at 90 °C leads to quite drastic changes in the morphology of the pyrolyzed products (Figure 3c). This process produces very small and round particles with diameters of about 0.6 µm with no visible pores on the surface (on the SEM length scale). Pretreatments with 0.2 M NaOH (Figure A3) lead to different effects after pyrolysis at 500 °C. The material now has clear edges and rougher surfaces on the particles including plenty of pores with maximum diameters of ca. 2.2 µm.

**Figure 4:**
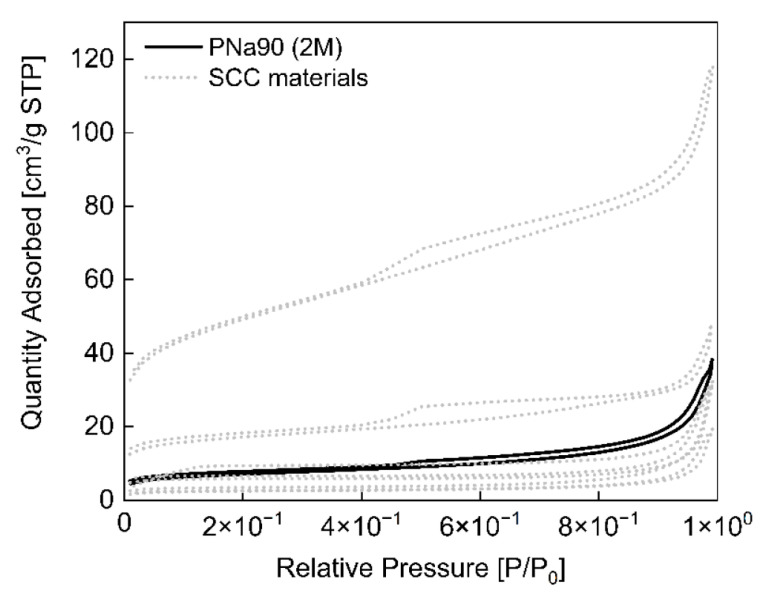
Nitrogen sorption isotherm of PNa90 (2 M) (black). Grey dotted lines are adsorption isotherms determined from SCC materials. These data are shown for comparison and will be discussed in detail in Section 3.2 below.

The nitrogen sorption isotherm of PNa90 (2 M) is displayed in Figure 4. The shape of the isotherm suggests that PNa90 (2 M) likely has a fairly large pore size distribution. The surface area calculated from the data is 24 m^2^/g. This is much smaller than the surface areas typically observed in commercial AC (up to 1500 m^2^/g). Similarly, the pore volume of 0.033 cm^3^/g is also much lower than commercial AC [1]. The pore diameter of the material is 9.9 nm.

### 3.2. Activated Carbons from Spent Coffee/Calcium Carbonate Mixtures

An alternative approach toward carbonaceous adsorbents using solid CaCO_3_ as activator was also explored. In this series, the SC powder was thoroughly mixed mechanically with dry calcium carbonate powder in a 1:1 (*w*/*w*) proportion. Pyrolysis was then done between 500 and 850 °C, i.e., below and above the point of decomposition of calcium carbonate at 825 °C. Table 2 shows the results from elemental analysis (EA) of these materials after washing. The washing process removes the CaCO_3_ and leaves the pure carbonaceous material behind, as evidenced from the EA data.

The IR spectra of these materials are shown in Figure 2b. Treatment temperatures up to 600 °C produce materials that exhibit IR spectra very similar to the spectra shown in Figure 2a. However, higher treatment temperatures of the SCD/calcium carbonate mixtures produce materials where the typical IR bands disappear and the corresponding IR spectra only show a straight line. This indicates that the vast majority of the surface groups vanishes during the heat treatment. Similar to the broadening effects observed in the IR spectra of PNa90 (2 M), a band broadening between 1500 and 1000 cm^−1^ is noticeable for SCC50 and SCC60 (Figure 2b). The spectrum of SCC65 shows an additional broad band around 3300 cm^−1^ which can be assigned to adsorbed water. However, as the pyrolysis temperature is increased, the resulting materials exhibit IR spectra where the bands around 1000 cm^−1^ are drastically reduced in intensity.

**Figure 5:**
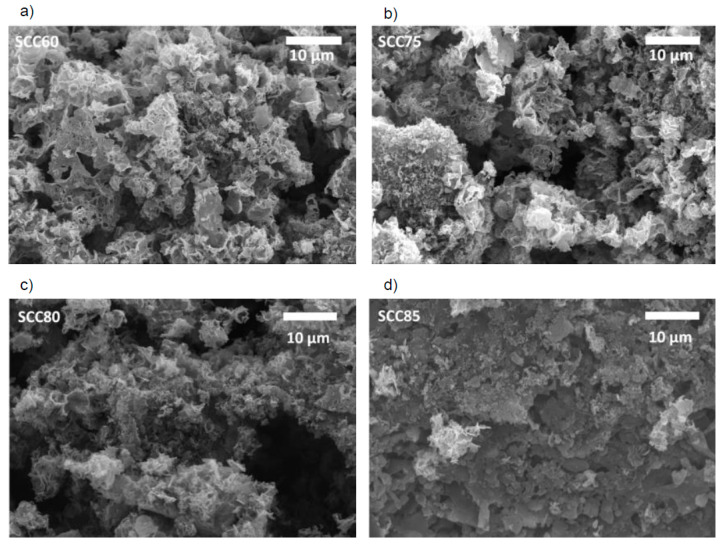
SEM images of samples (**a**) SCC60, (**b**) SCC75, (**c**) SCC80 and (**d**) SCC85.

Figure 5 shows representative SEM images of the SCC materials after washing out the calcium carbonate with aqueous hydrochloric acid. SEM clearly shows that the treatment temperature does not significantly affect the sample morphology. However, SEM also demonstrates that the calcium carbonate present in the reaction mixture leads to drastically different morphologies when compared, e.g., to the materials pretreated with NaOH (Figure 3).

The presence of the calcium carbonate leads to the formation of heavily interconnected flake-like carbons with a sponge-like appearance. The individual flakes are rather thin but are densely aggregated and tightly connected to neighboring flakes. This flake-based assembly produces a wide range of macropores that are clearly visible in the SEM (Figure 5). SEM also shows that the large, aggregated particles have a wide range of sizes and shapes, yet their architecture on the micrometer level is rather uniform and always constructed from the flaky building blocks just described.

The SEM images show no significant differences in the feature sizes or shapes vs. increasing carbonization temperatures. All powders contain particles with a broad size distribution and a variety of particle shapes (Figure 5). The mixture of SCD and calcium carbonate produces materials with a much finer substructure than the powders PNa90 (2 M) described above. The particles appear more “porous” (or appear to have a much rougher surface) and seem to have also broken down into smaller particles. Some particles show holes and larger open areas.

**Figure 6:**
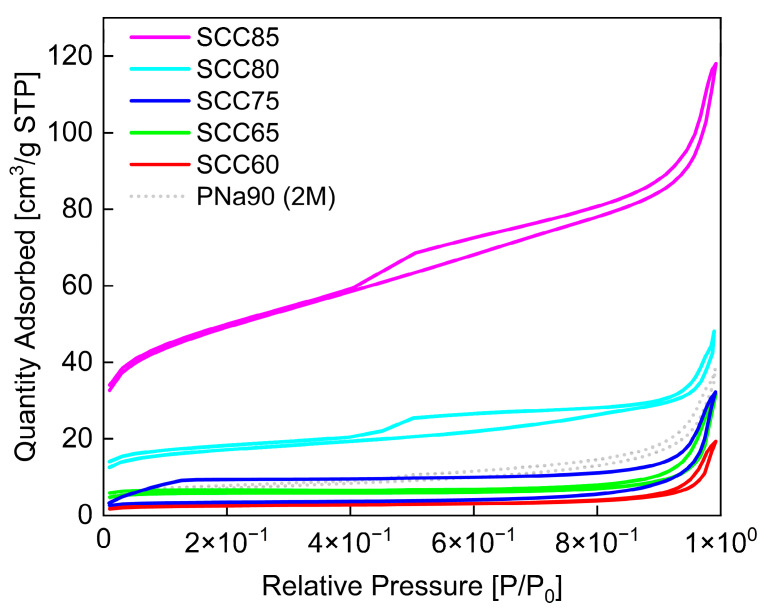
Nitrogen sorption isotherms of SCC materials (colored) and of PNa90 (2 M) (dotted gray). The data of the PNa90 (2 M) material are added for comparison; for details, see Section 3.1.

Figure 6 shows the corresponding nitrogen sorption data obtained from the SCC materials. All data indicate the presence of mesopores with a rather broad size distribution. Higher pyrolysis temperatures favor the formation of materials with higher surface areas with the highest surface area of ca. 166 m^2^/g obtained for the materials that were pyrolyzed at 850 °C. At the same time, the pore sizes decreases. Table 4 summarizes these results. Again, even the materials with the highest porosities are far from commercial AC in terms of both specific surface area and pore volume.

In summary, both groups of materials, PNa90 and SCC, show drastic property changes in comparison to the regular SC and its pyrolyzed form. A pyrolysis temperature of 500 °C is sufficient to break down the organic (carbohydrate, lipid, and protein) components to aromatic carbon (ssNMR, XRD), but does not lead to a complete removal of surface functional groups (ATR-IR). With increasing pyrolysis temperatures, more surface functional groups are removed from the materials, leaving behind an essentially carbon-only material. Both PNa90 and SCC materials exhibit an increased surface area compared to the original spent coffee (SEM, nitrogen sorption data). However, the surfaces obtained from BET analysis along with the corresponding total pore volume are still significantly smaller than those of commercial AC [1].

Clearly, the process of material synthesis is simple and effective; therefore, the materials were also investigated for the adsorption capabilities for dye removal from aqueous solution. This is a model case, and the removal of other substances can of course also be considered.

### 3.3. Dye Removal

Dye adsorption experiments were done with methylene blue (MB) and methyl orange (MO). MO and MB are common model compounds for dyestuff-polluted aqueous media because they are (1) water-soluble and (2) easy to detect and quantify via UV/Vis spectroscopy. As some dyes are health hazards [50,51,52,53], MO and MB are suitable model cases for dye-contaminated water.

**Figure 7:**
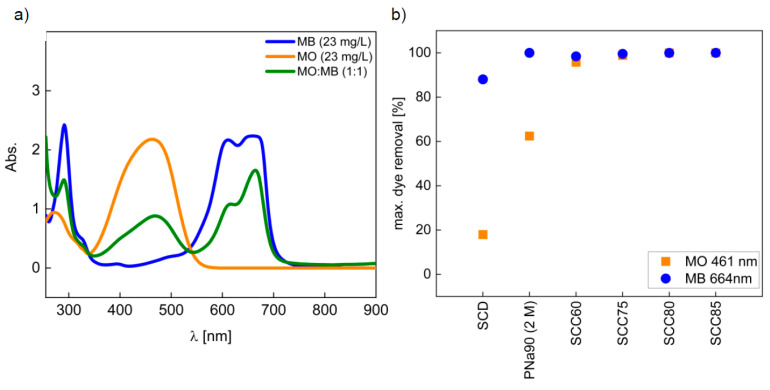
(**a**) Spectra of MB standard solution, MO standard solution, and MB/MO (1:1) mixed solution used for ad-sorption experiments. (**b**) MO and MB removal with washed adsorbents after 3 h of contact with the dye solution at room temperature.

Figure 7a shows the UV/Vis spectra of MB and MO solutions with 23 mg/L dye concentration along with the spectrum of a 1:1 MO/MB mixture. According to these spectra, the adsorption maxima of 664 nm for MB and 461 nm for MO were chosen for the comparison of dye uptake by the materials.

In a first study, PNa90 (2 M), which was washed neutral, and all as-prepared SCC materials were used for MB and MO removal in batches exposed to the solutions for 3 h. The SCC50 material (i.e., the material pyrolyzed at the lowest temperature of 500 °C) only removes below 20% of dye and was, therefore, not considered any further. It is noteworthy, however, that the non-pyrolyzed SCD raw material shows a remarkable adsorption capability for MB while it adsorbs nearly no MO. Similarly, PNa90 (2 M) shows a preferred adsorption of MB, but also takes up ca. 60% of the MO present in the solution. All other materials show a near-quantitative removal of both MO and MB (Figure 7b).

**Figure 8:**
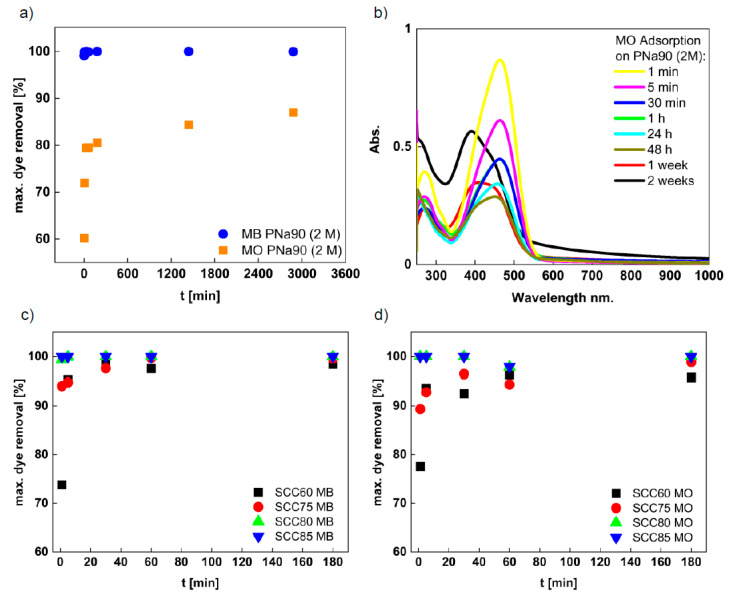
(**a**) Adsorption isotherms for MO and MB uptake with PNa90 (2M), (**b**) UV/Vis spectra of MO solu-tions vs. treatment times with PNa90 (2 M). (**c**) Adsorption isotherms of MB and (**d**) adsorption isotherms of MO uptake on SCC materials.

Figure 8 shows the adsorption isotherms (dye removal vs. treatment time at room temperature) for washed PNa90 (2 M) and SCC materials. Figure 8a shows the data for dye adsorption on PNa90 (2 M). Clearly, MB removal is faster and more effective. Already after 1 min, MB is removed almost completely from the aqueous phase. Quantitative (i.e., >99%) removal is achieved after 5 min and no re-release of MB into the solution is observed. In contrast, MO is taken up somewhat less effectively and the uptake is not as rapid. After an initial, rather fast uptake reaching ca. 80% MO removal, there is a second, much slower uptake regime starting at ca. 60 min. This slower uptake continues until the end of the experiment, but only contributes to a further uptake of ca. 6% after 48 h, resulting in an overall removal of around 86% of MO at the end of the process.

At this point, it is worthwhile to note that a significant hypsochromic shift is observed in the UV/Vis spectra of MO for longer adsorption times (Figure 8b). Already after 24 h, the spectra begin to show a peak broadening with a shoulder toward shorter wavelengths. This is further pronounced at longer treatment times of 48 h. Longer adsorption experiments running for 1 and 2 weeks show an even stronger change in the absorption bands, making a true quantification of the data obtained at long adsorption times difficult.

The reason for this hypsochromic shift is an increase in the pH value of the MO solution over time. As MO is a pH indicator, it reacts to this change in proton concentration via a color change. Indeed, measurements using a pH electrode show that the pH of the PNa90/MO mixed system starts out at pH 5 and increases to well above 7 after 24 h. This also indicates that, despite washing the material with 2 M HCl after pyrolysis, some NaOH remains in the material. The longer the material remains in the aqueous solution, the more NaOH seems to be released from the sample. This observation suggests that the current, prototype materials may need to be subjected to an improved cleaning process before application. On the other hand, such long batch exposure times are unrealistic for a larger-scale application, and we, therefore, performed further experiments at much shorter times.

Figure 8c,d show the adsorption isotherms obtained from the analogous experiments with the SCC materials obtained after removing the calcium carbonate. All SCC materials are effective adsorbents for MO and MB but there are differences. In the case of MB (Figure 8c), two different types of behavior can be distinguished: (1) SCC60 and SCC75 show a somewhat slower adsorption kinetics, and the final fraction of adsorbed dye is lower (around 98%) than in the case of SCC80 and SCC85. In contrast, (2) adsorption on SCC80 and SCC85 is very rapid and essentially reaches a quantitative removal of the dyes within 20 min.

A similar observation can be made for MO (Figure 8d). While the dye removal process was rather rapid and efficient, the SCC60 and SCC70 materials are again somewhat less effective than SCC80 and SCC85. In contrast to the data shown in Figure 7, these data show a slightly lower adsorption at around 60 min before returning to the very high fractions of removed dye at the end of the treatment. The reason for this slight “dip” is not clear at the moment and will be a matter of future research with materials improved from the current, first-generation adsorbents.

Overall, the SCC60 and SCC75 materials show a roughly quadratic increase in adsorption up to nearly 100%, resulting in a plateau. SCC80 and SCC85 essentially take up 100% of both dyes immediately after exposition, i.e., full dye removal is realized in less than 5 min. No significant adsorption/desorption fluctuation is noticeable in these materials.

These findings are especially interesting when compared to the analytical data described above. While PNa90 (2 M), SCC60, and SCC65 still show a significant presence of surface groups in the IR spectra (Figure 2), SCC75 through SCC85 show no more signals for any kind of surface groups. Hence, the observed dye adsorption can not be correlated to ionic interactions between material surfaces and type of dye. Rather, we currently speculate that the main interaction could be a van der Waals type interaction between the dye molecules and the adsorbent surface.

**Figure 9:**
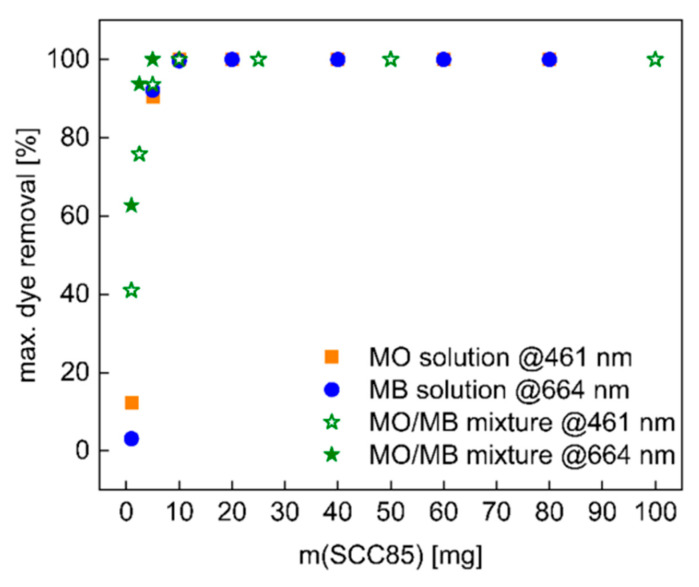
Study of mass dependence of SCC85 on adsorption of MO and MB (both 23 mg/L) and a mixture of both dyes (1:1 ratio). Samples were placed in 10 mL of solution for 3 h.

In addition to kinetic experiments, an evaluation of the effect of adsorbent dose was carried out with one of the best-performing materials, SCC85 (Figure 9). Overall, the data show that SCC85 is a highly effective adsorbent for both dyes with ≥90% of MO and MB taken up even at very low adsorbent doses of only 5 mg. Moreover, exposure to mixed solutions containing both dyes simultaneously has no impact on the adsorption capacity of the material. Again, all solutions are completely clear at the end of the treatment. In spite of this, MO adsorption is slightly faster at very low adsorbent doses of 1 and 2 mg. These two experiments also show a slight tint of the glassware, indicating that, at very low dosages, the sample environment may also have an influence, similar to a recent study on MO and MB uptake using a hydrogel/3D printed hybrid setup [50]. Adsorbent doses above 2 mg do not exhibit this behavior, indicating that the adsorbent dominates the removal process as intended.

## 4. Conclusions

Spent coffee is a cheap waste material that is produced daily in large amounts all over the world. It is thus a viable resource for numerous applications including low-cost water treatment. The current prototype study clearly shows that suitable treatments can improve the removal capacities for organic dyes from aqueous solution. Two different treatments are both highly promising: (1) pretreatment with NaOH followed by pyrolysis produces materials that favor the adsorption of MB, while (2) pyrolyzed mixtures of spent coffee and calcium carbonate powders produce materials that are almost indifferent to the charge of the dye molecule in solution. As a result, the simplest synthesis protocol (mixing spent coffee and calcium carbonate powders followed by pyrolysis) produces the most promising adsorbents. Simply by selecting the reaction temperature, the properties of the resulting materials can further be adjusted. Therefore, the current approach provides a truly sustainable approach toward low-cost water treatment materials. These materials are therefore quite similar to commercial activated carbons as far as their performance is considered. Interestingly, the data also show that the presence of active surface groups may not be necessary for good adsorption properties. The current, first-generation materials can further be upgraded by improving the cleaning processes, by improving the mixing ratio of the precursors leading to further improved surface areas and pore structures, and by implementing a true continuous (flow) process.

## Figures and Tables

**Table 1:** Overview of sample names and their preparation parameters. T_T_ is the temperature for the NaOH pre-treatment, while T_P_ is the pyrolysis temperature. For sample labels, see text above.

Label	Index ##	Pretreatment	T_T_ (°C)	T_P_ (°C)
SCD	-	-	-	-
PSC	50	-	-	500
PNa	90	NaOH (2 M)	90	500
SCC	50	dry CaCO_3_, mixing ratio 1:1 (*w*/*w*)	-	500
	60	dry CaCO_3_, mixing ratio 1:1 (*w*/*w*)		600
	65	dry CaCO_3_, mixing ratio 1:1 (*w*/*w*)		650
	75	dry CaCO_3_, mixing ratio 1:1 (*w*/*w*)		750
	80	dry CaCO_3_, mixing ratio 1:1 (*w*/*w*)		800
	85	dry CaCO_3_, mixing ratio 1:1 (*w*/*w*)		850

**Table 2:** Elemental analysis data in wt.% of dried spent coffee (SCD) and pyrolyzed samples either treated with 2 M NaOH (PNa) or mixed with CaCO_3_ (1:1) before pyrolysis (SCC). These are the materials that show significant dye uptake in the pretests. T_P_ is again the pyrolysis temperature.

	T_P_ (°C)	C (%)	H (%)	N (%)	S (%)
SCD	-	56.9	10.3	1.8	-
PNa90 (2 M)	500	41.0	0.6	0.9	0.6
SCC60	600	70.3	2.4	2.6	0.5
SCC75	750	82.0	1.6	2.4	0.5
SCC80	800	82.4	1.7	2.3	0.5
SCC85	850	82.2	1.4	2.5	0.4

**Table 3:** Classification of infrared bands [35,40,44,46,47,48,49]. Bands in the area of 2000 cm^−1^ represent the natural resonance of the diamond crystal used for measuring.

Wavenumber (cm^−1^)	Classification
3500–3300	O–H (stretching, intermolecular)
2930–2840	C–H (amorphous cellulose, caffeine), aromatic methoxyl and methylene groups, methyl groups
1740	C=O (stretching, esters)
1700–1600	caffeine, chlorogenic acids
1600–1300	C=C (aromatic compounds, oils, caffeine)
1430	C–H_2_ (crystallinity bond of cellulose)
1284–1240	C–O (aromatic ethers, esters, phenol)
1100–900	C–O (polysaccharides)
900–800	Β–glycosidic bond of cellulose

**Table 4:** BET surface, pore sizes, and pore volumes of the SCC materials determined from nitrogen sorption experiments.

Sample Name	BET Surface (m^2^/g)	Pore Size (Å)	Pore Volume (cm^3^/g)
SCC60	8.1	182.3	0.010
SCC65	17.9	236.6	0.015
SCC75	10.9	217.1	0.020
SCC80	56.1	63.1	0.035
SCC85	166.8	48.6	0.109

## Data Availability

All data are available from the authors upon request.

## References

[1] Cecen F., Aktas Ö. (2012). Activated Carbon for Water and Wastewater Treatment: Integration of Adsorption and Biological Treatment.

[2] Perrich J.R. (1981). Activated Carbon Adsorption for Wastewater Treatment.

[3] Marsh H., Rodríguez-Reinoso F. (2006). Activated Carbon.

[4] Saleem J., Shahid U.B., Hijab M., Mackey H., McKay G. (2019). Production and applications of activated carbons as adsorbents from olive stones. Biomass Conv. Bioref..

[5] Zhao H., Xue Y., Long L., Hu X. (2018). Adsorption of nitrate onto biochar derived from agricultural residuals. Water Sci. Technol..

[6] Unuabonah E.I., Nöske R., Weber J., Günter C., Taubert A. (2019). New micro/mesoporous nanocomposite material from low-cost sources for the efficient removal of aromatic and pathogenic pollutants from water. Beilstein J. Nanotechnol..

[7] Ucar S., Erdem M., Tay T., Karagöz S. (2009). Preparation and characterization of activated carbon produced from pomegranate seeds by ZnCl2 activation. Appl. Surf. Sci..

[8] Sivakumar B., Kannan C., Karthikeyan S. (2012). Preparation and characterization of activated carbon prepared from balsamodendron caudatum wood waste through various activation processes. Rasayan J. Chem..

[9] Rahman M.M., Awang M., Mohosina B.S., Kamaruzzaman B.Y., Nik W.W., Adnan C. (2012). Waste Palm Shell Converted to High Efficient Activated Carbon by Chemical Activation Method and Its Adsorption Capacity Tested by Water Filtration. APCBEE Procedia.

[10] Okman I., Karagöz S., Tay T., Erdem M. (2014). Activated Carbons from Grape Seeds By Chemical Activation With Potassium Carbonate And Potassium Hydroxide. Appl. Surf. Sci..

[11] Habeeb O.A., Ramesh K., Ali G.A., Yunus R.b.M. (2017). Low-cost and eco-friendly activated carbon from modified palm kernel shell for hydrogen sulfide removal from wastewater: Adsorption and kinetic studies. DWT.

[12] Gulipalli S., Prasad B., Wasewar K.L. (2011). Batch study, equilibrium and kinetics of adsorption of selenium using husk ash. J. Eng. Sci. Technol..

[13] Fidel R.B., Laird D.A., Spokas K.A. (2018). Sorption of ammonium and nitrate to biochars is electrostatic and pH-dependent. Sci. Rep..

[14] Das D., Samal D.P., BC M. (2015). Preparation of Activated Carbon from Green Coconut Shell and its Characterization. J. Chem. Eng. Process. Technol..

[15] Cuerda-Correa E.M., Diaz-Diez M.A., Macias-Garcia A., Ganan-Gomez J. (2006). Preparation of activated carbons previously treated with sulfuric acid. Appl. Surf. Sci..

[16] Caturla F., Molina-Sabio M., Rodríguez-Reinoso F. (1991). Preparation of Activated Carbon by Chemical Activation with ZnCl2. Carbon.

[17] Azevedo D.C., Araújo J.C.S., Bastos-Neto M., Torres A.E.B., Jaguaribe E.F., Cavalcante C.L. (2007). Microporous activated carbon prepared from coconut shells using chemical activation with zinc chloride. Microporous Mesoporous Mater..

[18] Kuppireddy S.K.R., Rashid K., Al Shoaibi A., Srinivasakannan C. (2014). Production and characterization of porous carbon from date palm seeds by chemical activation with H3PO4: Process optimization for maximizing adsorption of methylene blue. Chem. Eng. Commun..

[19] Karagöz S., Tay T., Ucar S., Erdem M. (2008). Activated carbons from waste biomass by sulfuric acid activation and their use on methylene blue adsorption. Bioresour. Technol..

[20] Biswas S., Siddiqi H., Meikap B.C., Sen T.K., Khiadani M. (2020). Preparation and Characterization of Raw and Inorganic Acid-Activated Pine Cone Biochar and Its Application in the Removal of Aqueous-Phase Pb2+ Metal Ions by Adsorption. Water Air Soil Pollut..

[21] Unuabonah E.I., Agunbiade F.O., Alfred M.O., Adewumi T.A., Okoli C.P., Omorogie M.O., Akanbi M.O., Ofomaja A.E., Taubert A. (2017). Facile synthesis of new amino-functionalized agrogenic hybrid composite clay adsorbents for phosphate capture and recovery from water. J. Clean. Prod..

[22] Unuabonah E.I., Günter C., Weber J., Lubahn S., Taubert A. (2013). Hybrid Clay: A New Highly Efficient Adsorbent for Water Treatment. ACS Sustain. Chem. Eng..

[23] Unuabonah E.I., Taubert A. (2014). Clay–polymer nanocomposites (CPNs): Adsorbents of the future for water treatment. Appl. Clay Sci..

[24] Liedtke A. (2019). Kaffeereport2019, Hamburg. https://www.tchibo.com/servlet/content/1302446/-/starteseite-deutsch/tchibo-unternehmen/sortiment/kaffee/kaffeereport/kaffeereport-2019.html;jsessionid=187477F94634FC8DBD370118F4B1A1A8.deliveryWorker.

[25] Iakovleva E., Sillanpää M., Maydannik P., Liu J.T., Allen S., Albadarin A.B., Mangwandi C. (2017). Manufacturing of novel low-cost adsorbent: Co-granulation of limestone and coffee waste. J. Environ. Manag..

[26] Cerino-Córdova F.J., Díaz-Flores P.E., García-Reyes R.B., Soto-Regalado E., Gómez-González R., Garza-González M.T., Bustamante-Alcántara E. (2013). Biosorption of Cu(II) and Pb(II) from aqueous solutions by chemically modified spent coffee grains. Int. J. Environ. Sci. Technol..

[27] Azouaoua N., Sadaouia Z., Mokaddema H. (2014). Removal of Lead from Aqueous Solution onto Untreated Coffee Grounds: A Fixed-bed Column Study. Chem. Eng. Trans..

[28] Azouaoua N., Sadaouia Z., Djaafrib A., Mokaddema H. (2010). Adsorption of cadmium from aqueous solution onto untreated coffee grounds: Equilibrium, kinetics and thermodynamics. J. Hazard. Mater..

[29] Franca A.S., Oliveira L.S., Ferreira M.E. (2009). Kinetics and equilibrium studies of methylene blue adsorption by spent coffee grounds. Desalination.

[30] Dávila-Guzmán N.E., de Jesús Cerino-Córdova F., Soto-Regalado E., Rangel-Mendez J.R., Díaz-Flores P.E., Garza-Gonzalez M.T., Loredo-Medrano J.A. (2013). Copper Biosorption by Spent Coffee Ground: Equilibrium, Kinetics, and Mechanism. Clean Soil Air Water.

[31] Gonçalves M., Guerreiro M.C., Ramos P.H., de Oliveira L.C.A., Sapag K. (2013). Activated carbon prepared from coffee pulp: Potential adsorbent of organic contaminants in aqueous solution. Water Sci. Technol..

[32] Ching S.L., Yusoff M.S., Aziz H.A., Umar M. (2011). Influence of impregnation ratio on coffee ground activated carbon as landfill leachate adsorbent for removal of total iron and orthophosphate. Desalination.

[33] Kemp K.C., Baek S.B., Lee W.-G., Meyyappan M., Kim K.S. (2015). Activated carbon derived from waste coffee grounds for stable methane storage. Nanotechnology.

[34] Lamine S.M., Ridha C., Mahfoud H.-M., Mouad C., Lotfi B., Al-Dujaili A.H. (2014). Chemical Activation of an Activated Carbon Prepared from Coffee Residue. Energy Procedia.

[35] Alves A.C.F., Antero R.V.P., Oliveira S.B.d., Ojala S.A., Scalize P.S. (2019). Activated carbon produced from waste coffee grounds for an effective removal of bisphenol-A in aqueous medium. Environ. Sci. Pollut. Res. Int..

[36] Kanai N., Yoshihara N., Kawamura I. (2019). Solid-state NMR characterization of triacylglycerol and polysaccharides in coffee beans. Biosci. Biotechnol. Biochem..

[37] Carruthers-Taylor T., Banerjee J., Little K., Wong Y.F., Jackson W.R., Patti A.F. (2020). Chemical Nature of Spent Coffee Grounds and Husks. Aust. J. Chem..

[38] Kögel-Knabner I. (2002). The macromolecular organic composition of plant and microbial residues as inputs to soil organic matter. Soil Biol. Biochem..

[39] Zhang D., Duan D., Huang Y., Yang Y., Ran Y. (2017). Composition and structure of natural organic matter through advanced nuclear magnetic resonance techniques. Chem. Biol. Technol. Agric..

[40] Taleb F., Ammar M., Mosbah M.B., Salem R.B., Moussaoui Y. (2020). Chemical modification of lignin derived from spent coffee grounds for methylene blue adsorption. Sci. Rep..

[41] Cheng C.-H., Lehmann J., Thies J.E., Burton S.D. (2008). Stability of black carbon in soils across a climatic gradient. J. Geophys. Res..

[42] Rashidi N.A., Yusup S., Ahmad M.M., Mohamed N.M., Hameed B.H. (2012). Activated Carbon from the Renewable Agricultural Residues Using Single Step Physical Activation: A Preliminary Analysis. APCBEE Procedia.

[43] Molina-Sabio M., Rodriguez-Reinoso F. (2004). Role of chemical activation in the development of carbon porosity. Colloids Surf. A Physicochem. Eng. Asp..

[44] Ballesteros L.F., Teixeira J.A., Mussatto S.I. (2014). Chemical, Functional, and Structural Properties of Spent Coffee Grounds and Coffee Silverskin. Food Bioprocess Technol..

[45] Gao G., Cheong L.-Z., Wang D., Shen C. (2018). Pyrolytic carbon derived from spent coffee grounds as anode for sodium-ion batteries. Carbon Resour. Convers..

[46] Ma X., Ouyang F. (2013). Adsorption properties of biomass-based activated carbon prepared with spent coffee grounds and pomelo skin by phosphoric acid activation. Appl. Surf. Sci..

[47] Kunusa W.R., Isa I., Laliyo L.A.R., Iyabu H. (2018). FTIR, XRD and SEM Analysis of Microcrystalline Cellulose (MCC) Fibers from Corncorbs in Alkaline Treatment. J. Phys. Conf. Ser..

[48] Hesas R.H., Arami-Niya A., Daud W.M.A.W., Sahu J.N. (2013). Preparation and Characterization of Activated Carbon from Apple Waste by Microwave-Assisted Phosphoric Acid Activation: Application in Methylene Blue. BioResources.

[49] Ciolacu D., Ciolacu F., Popa V.I. (2011). Amorphous Cellulose—Structure and Characterization. Cellul. Chem. Technol..

[50] Ihlenburg R.B.J., Lehnen A.-C., Koetz J., Taubert A. (2021). Sulfobetaine Cryogels for Preferential Adsorption of Methyl Orange from Mixed Dye Solutions. Polymers.

[51] Gillman P.K. (2011). CNS toxicity involving methylene blue: The exemplar for understanding and predicting drug interactions that precipitate serotonin toxicity. J. Psychopharmacol..

[52] Nejib A., Joelle D., Fadhila A., Sophie G., Malika T.-A. (2014). Adsorption of anionic dye on natural and organophilic clays: Effect of textile dyeing additives. DWT.

[53] Grassi M., Rizzo L., Farina A. (2013). Endocrine disruptors compounds, pharmaceuticals and personal care products in urban wastewater: Implications for agricultural reuse and their removal by adsorption process. Environ. Sci. Pollut. Res. Int..

